# Co-detection of antimicrobial resistance and virulence-associated determinants in *Staphylococcus aureus* isolated from radicular cysts in a post-disaster region of Türkiye

**DOI:** 10.1186/s12903-026-08449-6

**Published:** 2026-05-13

**Authors:** Ahmet CanHaskan, Nizami Duran, Elif Yaprak Colak, Fariz Selimli, Sibel Dagli

**Affiliations:** 1 Department of Oral and Maxillofacial Surgery, Faculty of Dentistry, Hatay Mustafa Kemal University, Antakya, Hatay 31060 Türkiye; 2 Department of Medical Microbiology, Faculty of Medicine, Hatay Mustafa Kemal University, Antakya, Hatay 31060 Türkiye

**Keywords:** *Staphylococcus aureus*, Antimicrobial resistance, Virulence genes, Radicular cysts, Post-disaster microbiology

## Abstract

**Background:**

Radicular cysts are among the most common chronic odontogenic lesions, yet the molecular mechanisms of resistance and virulence of their microbial flora remain incompletely defined. Following the 2023 earthquakes in Hatay, Türkiye, medical facilities were severely disrupted, creating a post-disaster clinical setting. This cross-sectional, isolate-based study descriptively evaluated antimicrobial resistance phenotypes and selected virulence-associated genes among *S. aureus* isolates recovered from radicular cyst infections in this setting.

**Methods:**

A total of 225 clinical *S. aureus* isolates were recovered from surgically excised radicular cysts. Antimicrobial susceptibility testing was performed by disk diffusion according to CLSI guidelines. Eleven selected targets, including *mecA* and virulence-associated genes (*hla, hlb, pvl, fnbA, fnbB, clfA, cna, ebpS, bbp*), together with 16S rRNA for species confirmation, were detected by PCR. Pairwise and higher-level co-occurrence analyses were used to describe gene co-detection patterns. Associations between resistance categories and gene positivity were evaluated using Fisher’s exact test or chi-square test, as appropriate.

**Results:**

Resistance to one, two, and three or more antimicrobial classes was observed in 45.3%, 32.9%, and 21.8% of isolates, respectively. *MecA* positivity was more frequent in isolates with higher resistance categories, increasing from 4.9% in single-class-resistant isolates to 30.6% in multidrug-resistant (MDR) isolates (*p* < 0.001). Among the selected virulence-associated genes, *fnbA* (58.7%), *hla* (52.9%), and *cna* (45.8%) were most frequently detected. MDR isolates showed higher detection frequencies of *hla* (85.7%), *fnbA* (81.6%), and *cna* (73.5%) than lower resistance categories. Although *pvl* was detected in only 8.0% of isolates overall, it was more frequently detected among MDR isolates. Co-occurrence analysis identified recurrent gene co-detection patterns involving adhesion- and hemolysin-associated genes, most commonly *fnbA-cna, hla-fnbA*, and *hla-cna*. Higher-order gene combinations (≥ 4 genes) were uncommon and reflected isolates that simultaneously carried multiple virulence-associated genes.

**Conclusions:**

In this isolated collection, multidrug resistance was associated with more frequent detection of selected virulence-associated genes in cyst-derived *S. aureus* isolates. The results may be relevant for the combined use of phenotypic susceptibility testing and molecular detection of major resistance markers, particularly *mecA*, in laboratory characterization of isolates. Because the study lacked a pre-disaster comparator and external control group, the recorded patterns cannot be attributed to the disaster context; rather, they provide descriptive baseline data from a post-disaster clinical setting.

**Supplementary Information:**

The online version contains supplementary material available at 10.1186/s12903-026-08449-6.

## Introduction

Radicular cysts develop primarily as a consequence of pulpal necrosis and long-standing periapical inflammation, and their biological behavior is closely influenced by the microbial diversity within the lesion [6, 7]. Although *Staphylococcus aureus* is not typically regarded as a primary etiological agent in endodontic or periapical disease, its detection in radicular cysts is clinically relevant due to its notable virulence and antimicrobial resistance. In lesions that fail to resolve with conventional treatment or tend to recur, the ability of certain microorganisms to form biofilms and persist in hostile microenvironments becomes particularly important. *S. aureus* is one such organism; its biofilm-forming potential and adaptability to diverse environmental conditions suggest a possible role in refractory or persistent cystic infections. Furthermore, mechanistic studies examining factors that modulate *S. aureus* susceptibility to antimicrobial agents highlight how intrinsic and acquired resistance mechanisms may complicate management when the organism is present [11]. For these reasons, characterizing the virulence determinants and antimicrobial resistance profiles of *S. aureus* recovered from radicular cysts is essential to understand its pathogenic significance better and to inform more effective diagnostic and therapeutic strategies. Unlike obligate anaerobic endodontic pathogens, *S. aureus* possesses a broad repertoire of resistance and virulence determinants, making it a clinically relevant opportunistic pathogen when detected in persistent lesions.

Recent studies have increasingly highlighted the clinical importance of *S. aureus* in oral and endodontic infections. Although odontogenic cysts harbor diverse microbial communities, *S. aureus* has been increasingly detected in oral reservoirs, with several epidemiological studies from the United States reporting rising detection rates of *S. aureus* in dental and orofacial infections over the past decade [18, 27, 28]. Reports from Europe have documented Staphylococcus aureus-positive odontogenic lesions associated with complications including abscess formation, sinus tract development, and delayed tissue healing. These findings underscore the clinical significance of S. aureus in odontogenic lesions and warrant further investigation into its distribution and molecular characteristics in radicular cysts. As a common colonizer of skin and mucosal surfaces [27], *S. aureus* has multiple opportunities to enter the oral cavity, and its recovery from endodontic infections has frequently been linked with treatment failure, persistent inflammation, and recurrence. Given its robust virulence repertoire and the growing concern about antibiotic resistance, a detailed investigation of the genetic and phenotypic characteristics of *S. aureus* in radicular cysts is essential to understand its clinical impact and inform more effective management strategies [3, 27].

To better understand the pathogenic potential of *S. aureus* in radicular cysts, we focused on a group of virulence genes involved in adhesion, matrix binding, and toxin production. Adhesion factors such as *clfA* and *fnbA/B* encode surface proteins that interact with fibrinogen and fibronectin, allowing the organism to anchor to host tissues and establish a stable foothold within the lesion [9, 23, 25]. In addition, structural binding proteins encoded by *bbp, ebpS*, and *cna* facilitate attachment to bone sialoprotein, elastin, and collagen. These interactions enhance biofilm development and help the bacteria evade immune clearance, features commonly associated with long-standing or recurrent infections [19, 30]. Virulence is further reinforced by toxin-related genes such as *hla, hlb*, and *pvl*, which encode cytolytic factors that damage epithelial and immune cells and drive strong inflammatory responses [1, 24]. When these determinants are co-detected, they may indicate strains carrying multiple adhesion- and toxin-associated traits. Nevertheless, it is important to note that the present study does not evaluate their direct clinical or functional consequences.

## Materials and methods

### Sample collection and bacterial isolation

This study was designed as a cross-sectional, observational, isolate-based molecular epidemiology study aimed at descriptively characterizing antimicrobial resistance phenotypes and virulence gene distributions in *S. aureus* isolates recovered from radicular cyst infections. A total of 225 *Staphylococcus aureus* isolates were obtained from radicular cyst lesions excised during surgical enucleation procedures performed at the Mustafa Kemal University Faculty of Dentistry Hospital. To reduce potential confounding effects on microbial composition, patients who had received systemic antibiotic therapy within the preceding four weeks, as well as those with documented immunodeficiency, were excluded from the study.

All surgical procedures were performed under strictly controlled aseptic conditions to minimize potential contamination originating from the oral cavity or skin flora.

Prior to surgical incision, the oral mucosa and surrounding operative field were disinfected using standard antiseptic protocols, including 0.2% chlorhexidine gluconate or povidone-iodine, in accordance with routine oral surgery practice at our center.

Following surgical exposure, superficial cystic tissue and overlying epithelial layers were carefully removed and discarded. Microbiological sampling was restricted to the deep intralesional region of the cyst cavity, with deliberate avoidance of contact with the oral mucosa. For this purpose, cyst wall tissue fragments and, when appropriate, intralesional aspirates were collected instead of surface swab samples, as these materials were considered more representative of the intracystic microbial environment. Collected specimens were placed into sterile containers immediately after sampling and transported to the microbiology laboratory under cold-chain conditions without delay. Primary bacterial isolation was performed by inoculating the samples onto mannitol salt agar (MSA) and 5% sheep blood agar plates, then incubating aerobically at 37 °C for 24 h.

Phenotypic characteristics consistent with *S. aureus* were further evaluated using standard microbiological methods, including colony morphology assessment, Gram staining, catalase testing, and coagulase testing. Definitive identification of *S. aureus* isolates was performed using the VITEK 2 Compact automated identification system.

### Ethics approval and consent to participate

This study was approved by the Hatay Mustafa Kemal University Tayfur Ata Sökmen Faculty of Medicine Clinical Research Ethics Committee (IRB Approval Code: 2023/16, Decision No: 07, Date: 03 September 2023). All procedures involving human participants were conducted in accordance with the ethical standards of the institutional research committee and with the Declaration of Helsinki. Written informed consent was obtained from all participants prior to sample collection.

### Antimicrobial susceptibility testing

Susceptibility to all listed antimicrobial agents was assessed using the disk diffusion method on Mueller–Hinton agar, in accordance with CLSI guidelines. Antibiotic susceptibility profiles were determined according to the standards published by the Clinical and Laboratory Standards Institute [5]. The disk diffusion method was applied on Mueller–Hinton agar to evaluate the susceptibility of *S. aureus* isolates to 16 antimicrobial agents. The antibiotics tested were: penicillin G (10 U), amoxicillin (10 µg), amoxicillin-clavulanic acid (20/10 µg), cefoxitin (30 µg), azithromycin (15 µg), ciprofloxacin (5 µg), moxifloxacin (5 µg), clindamycin (2 µg), doxycycline (30 µg), minocycline (30 µg), chloramphenicol (30 µg), trimethoprim–sulfamethoxazole (1.25/23.5 µg), gentamicin (10 µg), linezolid (30 µg), rifampicin (5 µg), and vancomycin (30 µg). Bacterial inocula were prepared by suspending fresh colonies in sterile PBS (phosphate-buffered saline). Bacterial density was adjusted to a turbidity equivalent to 0.5 McFarland standard. Plates were incubated at 37 °C for 18–24 h. Methicillin resistance was assessed by cefoxitin testing and confirmed molecularly by polymerase chain reaction (PCR) of the *mecA* gene. *S. aureus* ATCC 25923 was used as a quality control strain throughout susceptibility testing. According to international definitions, multidrug resistance (MDR) is defined as resistance to one or more agents from at least three different antimicrobial classes [5]. All listed antimicrobial agents were tested using the disk diffusion method in accordance with CLSI M100 guidelines. Minimum inhibitory concentration (MIC) testing was not performed except where CLSI interpretive criteria required confirmatory evaluation.

### DNA extraction and PCR amplification

Genomic DNA was extracted from overnight cultures grown in tryptic soy broth using the Qiagen DNeasy Blood & Tissue Kit (Hilden, Germany) according to the manufacturer’s instructions. DNA concentration and purity were assessed spectrophotometrically using a NanoDrop instrument. Extracted DNA was stored at −20 °C until analysis.

PCR amplification was conducted to detect eleven target genes. These genes are classified into functional categories: species confirmation (16S rRNA), antibiotic resistance gene (*mecA*), adhesion and binding proteins (*clfA, fnbA, fnbB, bbp, ebpS, cna*), and cytolytic/virulence toxins (*hla, hlb, pvl*) [13–15, 21]. Primer sequences and expected product sizes are provided in Table 1.

**Table 1:** Primer sequences used for amplification of virulence and resistance genes in *Staphylococcus aureus*

Target gene	Primer sequence (5′–3′)	Amplicon size (bp)	Reference
16S rRNA	F: CCTATAAGACTGGGATAACTTCGGG R: CTTTGAGTTTCAACCTTGCGGTCG	791	[16]
clfA	F: GTAGGTACGTTAATCGGTT R: CTCATCAGGTTGTTCAGG	638	[21]
mecA	F: TCCAGGAATGCAGAAAGACCAAAGC R: GACACGATAGCCATCTTCATGTTGG	331	[14]
hla	F: CTGATTACTATCCAAGAAATTCGATTG R: CTTTCCAGCCTACTTTTTTATCAG	209	[8]
hlb	F: GTGCACTTACTGACAATAGTGC R: GTTGATGAGTAGCTACCTTCAGT	309	[8]
fnbB	F: CACAACCAGCAAATATAG R: CTGTGTGGTAATCAATGTC	1362	[21]
fnbA	F: GTAACAGCTAATGGTCGAATTGATACT R: CAAGTTCGATAGGAGTACTATGTTC	524	[21]
bbp	F: CAGTAAATGTGTCAAAAGA R: TACACCCTGTTGAACTG	575	[21]
ebpS	F: CAATCGATAGACACAAATTC R: CAGTTACATCATCATGTTTA	186	[21]
cna	F: ATATGAATTCGAGTATAAGGAGGGGTT R: ATTCTGCAGAGAACTAAGAATAGCCTT	423	[20]
pvl (luk-PVL)	F: ATCATTAGGTAAAATGTCTGGACATGATCCA R: GCATCAASTGTATTGGATAGCAAAAGC	433	[13]

Each PCR reaction was carried out in a 25 µL volume. The mix contained 2.5 µL of 10 × PCR buffer, 2.0 mM MgCl_2_, 0.2 mM of each dNTP, and 1 µM of each forward and reverse primer. It also included 1 U Taq DNA polymerase and approximately 50 ng of template DNA. Thermocycling was performed in a programmable thermal cycler under these conditions: initial denaturation at 94 °C for 5 min; 35 cycles of denaturation at 94 °C for 30 s, gene-specific annealing temperature for 30 s, and extension at 72 °C for 1 min; followed by a final extension at 72 °C for 7 min. PCR products were resolved on 1.5% agarose gels with ethidium bromide and viewed via UV transilluminator. A 100 bp DNA ladder was used as a marker to verify amplicon sizes [13, 14, 21]. All genes were amplified under identical cycling conditions using singleplex PCR reactions. Multiplex PCR was not performed.

### Data analyses

Data analyses were performed using GraphPad Prism version 10 (GraphPad Software, San Diego, CA, USA). Statistical analyses primarily focused on categorical variables, including antimicrobial resistance categories and the presence or absence of resistance and virulence-associated genes. Descriptive statistics were used to summarize antimicrobial resistance patterns and gene distributions. Categorical comparisons were conducted using the chi-square test or Fisher’s exact test, as appropriate, depending on expected cell counts. Analyses were restricted to categorical comparisons. No formal correction for multiple comparisons was applied; therefore, reported *p*-values represent unadjusted two-tailed tests. All tests were two-tailed, and a *p* value < 0.05 was considered statistically significant.

A formal a priori sample size calculation was not performed. The study was designed as a cross-sectional, isolate-based descriptive molecular epidemiology investigation based on the number of eligible *S. aureus* isolates recovered during the study period. Accordingly, the findings should be interpreted as descriptive, with limited statistical power, particularly for infrequently detected genes and higher-order gene combinations.

## Results

A total of 225 *Staphylococcus aureus* isolates were obtained from radicular cyst specimens. Antimicrobial susceptibility testing revealed that 45.3 percent of isolates were resistant to a single antibiotic class, 32.9 percent to two classes, and 21.8 percent to three or more antibiotic classes (multidrug resistance, MDR) (Table 2). Initial confirmation of isolate identity was verified by 16S rRNA PCR (Supplementary Figure S1).

**Table 2:** Prevalence of individual virulence and resistance genes among *S. aureus* isolates (*n* = 225)

Gene	Positive (n)	Positivity (%)
*16S rRNA*	225	100.0
*hla*	119	52.9
*hlb*	59	20.9
*fnbA*	132	58.7
*clfA*	71	25.3
*cna*	103	45.8
*ebpS*	35	12.4
*pvl*	18	8.0
*mecA*	28	12.4
*fnbB*	19	8.4
*bbp*	13	5.8

Overall, *mecA* positivity was 12.4%, indicating moderate methicillin resistance among oral isolates (Supplementary Figure S2). *MecA* positivity was more frequent with increasing resistance category, rising from 4.9 percent in single-class-resistant isolates to 10.8 percent in dual-resistant isolates and 30.6 percent in MDR isolates. MRSA was defined as *mecA*-positive isolates and was analyzed within the existing resistance categories (single-class, dual-class, and MDR) rather than as a separate resistance group. Thus, *mecA*-positive isolates could be present in any resistance category depending on the number of antimicrobial classes to which they were resistant.

**Fig. 1:**
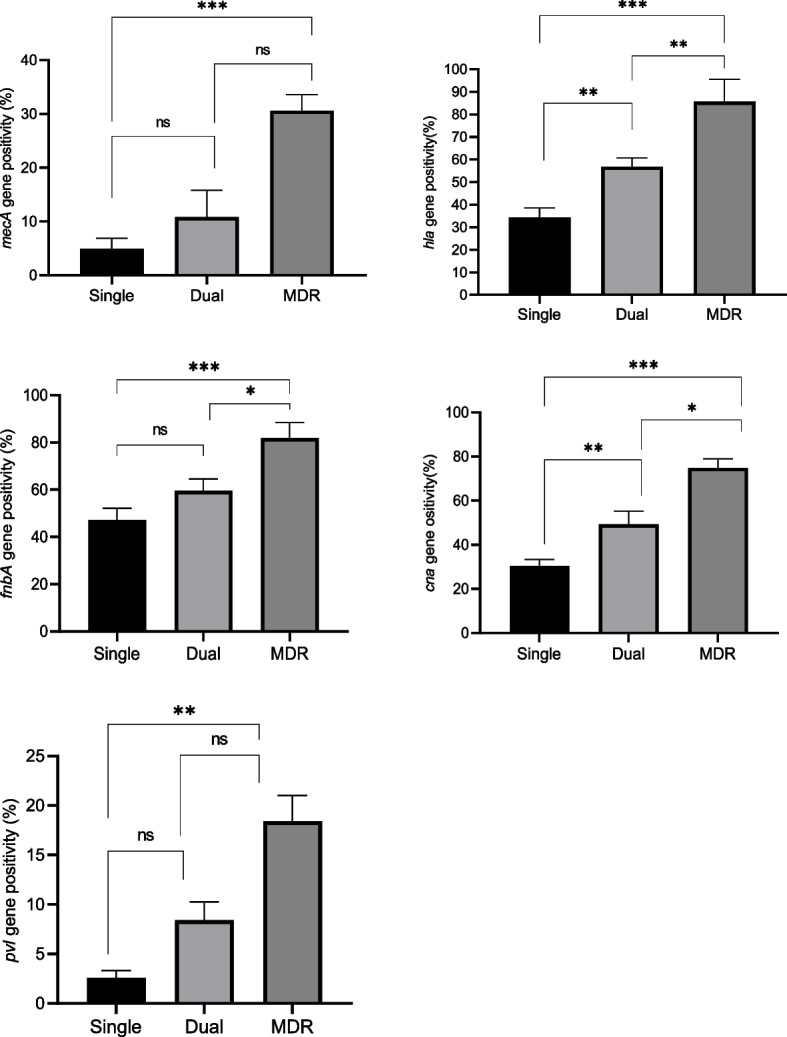
Distribution of virulence and resistance-associated gene positivity across antimicrobial resistance levels in *S. aureus* isolates. Figure legend: The positivity rates of *mecA, hla, fnbA, cna*, and *pvl* genes were compared among single-class resistant, dual-class resistant, and multidrug-resistant (MDR) isolates using Fisher’s exact test (two-tailed). For *mecA*, a highly significant difference was observed between single-class and MDR isolates (*p* < 0.001), whereas comparisons between single vs dual (*p* = 0.216) and dual vs MDR (*p* = 0.051) were not statistically significant. The *hla* gene demonstrated significant differences across all pairwise comparisons (single vs dual, *p* = 0.006; single vs MDR, *p* < 0.001; dual vs MDR, *p* = 0.001). For *fnbA*, the difference was significant between single vs MDR (*p* < 0.001) and dual vs MDR (*p* = 0.019), while single vs dual did not reach statistical significance (*p* = 0.114). Similarly, *cna* positivity differed significantly between single vs dual (*p* = 0.006), single vs MDR (*p* < 0.001), and dual vs MDR (*p* = 0.031). For *pvl*, a significant difference was detected only between single vs MDR isolates (*p* = 0.002), whereas other comparisons were not significant (single vs dual, *p* = 0.149; dual vs MDR, *p* = 0.071). Error bars represent SEM. Statistical significance is indicated on the graph (*p < 0.05; **p < 0.01; ***p < 0.001; ns, not significant)

Pairwise comparisons using Fisher’s exact test (two-tailed) showed that *mecA* positivity differed significantly between single-class and MDR isolates (*p* < 0.001), whereas single vs dual (*p* = 0.216) and dual vs MDR (*p* = 0.051) were not significant (Fig. 1).

When virulence gene distribution was examined, the most prevalent genes were *fnbA* (58.7 percent), *hla* (52.9 percent), and *cna* (45.8 percent), whereas *pvl* (8.0 percent), *fnbB* (8.4 percent), and *bbp* (5.8 percent) were detected at lower frequencies (Table 2). Approximately 70% of isolates carried at least one adhesion-associated gene (*fnbA, clfA, cna, ebpS, fnbB*, or *bbp*). *ClfA* was detected in 25.3% of isolates but did not show a statistically significant association with resistance category. Notably, *hla, fnbA*, and *cna* showed markedly higher positivity in MDR isolates (85.7 percent, 81.6 percent, and 73.5 percent, respectively; *p* < 0.05), indicating that adhesion- and toxin-related genes were more frequently detected among isolates with higher resistance levels (Table 3).

**Table 3:** Distribution of *mecA* and virulence genes according to antibiotic resistance levels in *S. aureus* isolates from radicular cyst infections (*n* = 225)

Resistance level	Isolates n (%)	mecA (+) n (%)	hla (+) n (%)	fnbA (+) n (%)	cna (+) n (%)	pvl (+) n (%)	fnbB (+) n (%)	bbp (+) n (%)
Single-class resistant	102 (45.3%)	5 (4.9%)	35 (34.3%)	48 (47.1%)	31 (30.4%)	3 (2.9%)	8 (7.8%)	6 (5.9%)
Co-resistant (dual-class)	74 (32.9%)	8 (10.8%)	42 (56.8%)	44 (59.5%)	36 (48.6%)	6 (8.1%)	6 (8.1%)	4 (5.4%)
MDR (multidrug resistant)	49 (21.8%)	15 (30.6%)	42 (85.7%)	40 (81.6%)	36 (73.5%)	9 (18.4%)	5 (10.2%)	3 (6.1%)

*Hla* positivity increased across resistance categories and was significant in all pairwise comparisons (single vs dual, *p* = 0.006; single vs MDR, *p* < 0.001; dual vs MDR, *p* = 0.001) (Fig. 1, Supplementary Figure S3).

*FnbA* positivity showed significant differences between single vs MDR (*p* < 0.001) and dual vs MDR (*p* = 0.019), while single vs dual was not significant (*p* = 0.114) (Fig. 1, Supplementary Figure S4).

*Pvl* positivity was significantly higher in MDR compared with single-class isolates (*p* = 0.002), whereas single vs dual (*p* = 0.149) and dual vs MDR (*p* = 0.071) were not significant (Fig. 1, Supplementary Figure S5).

**Fig. 2:**
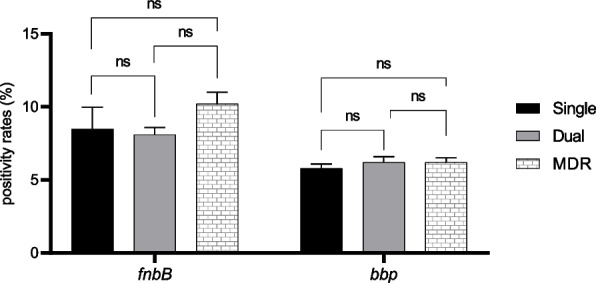
Positivity of additional adhesion-related genes (*fnbB* and *bbp*) according to antimicrobial resistance level in *S. aureus* isolates. Figure legend: The distribution of *fnbB* and bbp genes was compared across single-class resistant, dual-class resistant, and MDR isolates using Fisher’s exact test (two-tailed). No statistically significant differences were observed for fnbB (single vs dual, *p* = 1.000; single vs MDR, *p* = 0.579; dual vs MDR, *p* = 0.744) or bbp (all pairwise comparisons, *p* = 1.000). These findings suggest that not all adhesion-associated determinants are linked to increasing antimicrobial resistance. Error bars represent SEM

*FnbB* showed no significant association with resistance category (single vs dual, *p* = 1.000; single vs MDR, *p* = 0.579; dual vs MDR, *p* = 0.744) (Fig. 2, Supplementary Figures S6).

*Bbp* positivity did not differ across resistance categories (all pairwise comparisons, *p* = 1.000) (Fig. 2, Supplementary Figure S7).

*Cna* positivity increased substantially with resistance severity and was significant in all pairwise comparisons (single vs dual, *p* = 0.006; single vs MDR, *p* < 0.001; dual vs MDR, *p* = 0.031) (Fig. 1, Supplementary Figure S8).

*Hlb* positivity (20.9%) did not demonstrate a statistically significant association with resistance category across pairwise comparisons (Fig. 2, Supplementary Figure S9).

Similarly, *ebpS* (12.4%) showed no significant difference across resistance levels (Fig. 2, Supplementary Figure S10).

Pairwise and higher-order co-occurrence analysis identified the most common gene combinations: *fnbA-cna* (31.1 percent), *hla-fnbA* (28.0 percent), and *hla-cna* (24.0 percent). These represent recurrent co-detection patterns of adhesion- and hemolysin-associated genes (Table 4).

**Table 4:** Co-occurrence patterns of virulence genes among *S. aureus* isolates (*n* = 225)

Cluster Order	Gene Combination	n	%
Pairwise (2-gene)	*fnbA* + *cna*	88	31.1
	*hla* + *fnbA*	79	28.0
	*hla* + *cna*	68	24.0
	*fnbA* + *clfA*	64	22.7
	*clfA* + *cna*	50	17.8
	*hla* + *clfA*	46	16.4
	*hlb* + *fnbA*	40	14.2
	*hlb* + *cna*	35	12.4
	*hla* + *hlb*	31	11.1
	*hlb* + *clfA*	25	8.9
	*fnbA* + *ebpS*	24	8.4
	*cna* + *ebpS*	16	5.8
	*fnbA* + *pvl*	14	4.9
	*hla* + *ebpS*	14	4.9
	*clfA* + *ebpS*	11	4.0
	*hlb* + *ebpS*	10	3.6
	*cna* + *pvl*	9	3.1
	*hla* + *pvl*	8	2.7
	*clfA* + *pvl*	5	1.8
	*hlb* + *pvl*	4	1.3
	*ebpS* + *pvl*	3	0.9
Triple (3-gene)	*hla* + *fnbA* + *cna*	61	21.8
	*fnbA* + *clfA* + *cna*	46	16.4
	*hla* + *fnbA* + *clfA*	36	12.9
	*hla* + *clfA* + *cna*	34	12.0
	*hlb* + *fnbA* + *cna*	23	8.0
	*hla* + *fnbA* + *ebpS*	13	4.4
	*fnbA* + *cna* + *ebpS*	11	4.0
	*hlb* + *fnbA* + *clfA*	9	3.1
	*hla* + *cna* + *ebpS*	9	3.1
	*hla* + *fnbA* + *pvl*	6	2.2
Quadruple (4-gene)	*hla* + *fnbA* + *clfA* + *cna*	30	10.7
	*hla* + *hlb* + *fnbA* + *cna*	19	6.7
	*fnbA* + *clfA* + *cna* + *ebpS*	9	3.1
	*hla* + *fnbA* + *cna* + *ebpS*	8	2.7
	*hla* + *fnbA* + *clfA* + *ebpS*	6	2.2
	*hla* + *fnbA* + *clfA* + *pvl*	4	1.3
	*hlb* + *fnbA* + *clfA* + *cna*	4	1.3
	*fnbA* + *clfA* + *cna* + *pvl*	3	0.9
Quintuple (5-gene)	*hla* + *hlb* + *fnbA* + *clfA* + *cna*	14	4.9
	*hla* + *fnbA* + *clfA* + *cna* + *ebpS*	8	2.7
	*hla* + *fnbA* + *clfA* + *cna* + *pvl*	4	1.3
	*hlb* + *fnbA* + *clfA* + *cna* + *ebpS*	3	0.9
	*hla* + *fnbA* + *clfA* + *ebpS* + *pvl*	1	0.4
High-order (≥ 6-gene)	*hla* + *hlb* + *fnbA* + *clfA* + *cna* + *ebpS*	5	1.8
	*hla* + *hlb* + *fnbA* + *clfA* + *cna* + *pvl*	3	0.9
	*hla* + *hlb* + *fnbA* + *clfA* + *cna* + *ebpS* + *pvl*	1	0.4

Triple-gene clusters followed similar patterns, with *hla* + *fnbA* + *cna* (21.8 percent) being the most frequent combination (Table 4).

Quadruple and quintuple gene clusters showed decreasing frequency with increasing combination size, consistent with biological expectations. High-order clusters (≥ 6 genes), although rare, represented isolates carrying multiple virulence-associated genes simultaneously (Table 4).

Taken together, Figs. 1 and 2 and Tables 1, 2, 3 and 4 show that selected virulence-associated genes (*hla, fnbA, cna, pvl*) were more frequently detected among MDR isolates, whereas *fnbB* and bbp were not significantly associated with resistance category. *MecA* positivity was also more frequent among MDR isolates.

## Discussion

The observed coexistence of antimicrobial resistance and virulence-associated genes in the present study should be interpreted as a descriptive finding within a post-disaster setting rather than as evidence of disaster-driven amplification. Given the cross-sectional design and the absence of a comparator group, no causal or temporal conclusions regarding the impact of healthcare disruption can be drawn.

*Staphylococcus aureus* isolates from radicular cyst infections in Hatay, Türkiye, showed substantial antimicrobial resistance. These isolates carried multiple virulence- and resistance-associated genes. This coexistence is a descriptive finding in the studied isolate population and may be relevant to the epidemiological characterization of oral *S. aureus* isolates [12, 18].

Detection of *mecA* occurred in 12.4 percent of isolates. Its presence increased from single-class to MDR isolates (4.9–30.6 percent). This pattern aligns with global evidence linking *mecA* carriage to the expansion of resistance phenotypes [28]. The statistically significant distribution of *mecA* positivity across resistance categories indicates that *mecA* was more frequently detected among isolates with broader resistance phenotypes (Fig. 1). Although selective antibiotic pressure is one possible explanation discussed in the literature, the present study design does not permit mechanistic or causal inference. Although the observed *mecA* positivity rate is lower than the 26.7 percent previously reported in the literature, the correlation with resistance categories remains strong [4]. In addition to *mecA*, alternative methicillin resistance determinants such as *mecC* have been described in *S. aureus*; however, *mecC* was not investigated in the present study [2].

Previous reports have described oral S. aureus detection in endodontic infections in both Türkiye and elsewhere [3, 27]. However, those studies use different designs, populations, specimen types, and outcome measures. This limits direct comparison with the present isolate-based dataset. In the current cohort, 21.8 percent of isolates met the MDR definition. No pre-disaster comparator or external control group was included. These findings are descriptive observations from a post-disaster clinical setting, not evidence of a disaster-related increase. However, the cross-sectional design does not allow temporal comparison or causal inference regarding the impact of healthcare disruption on resistance patterns. Although post-disaster environments have been associated with disruptions in healthcare delivery in previous reports [10, 17, 29], the present study does not allow attribution of the observed patterns to this context.

Gene profiling further showed a high prevalence of adhesion-associated determinants, including *fnbA* (58.7 percent), *cna* (45.8 percent), and *clfA* (25.3 percent), which aligns with global trends in oral and skin *S. aureus* [18]. Notably, *fnbA* and *cna* were more frequently detected among MDR isolates (Fig. 1). Because these genes have been implicated in adhesion in previous literature [22], their co-detection in resistant isolates may be relevant to persistence; however, colonization capacity, biofilm behavior, and clinical persistence were not directly assessed in the present study. These findings should be interpreted as descriptive observations within a post-disaster setting rather than evidence of disaster-driven amplification. The study focused on selected adhesion and toxin-associated genes previously reported in oral *S. aureus* isolates; biofilm-regulatory genes such as *icaA* were beyond the predefined scope of this investigation.

Hemolysin genes showed similarly notable patterns. *Hla* (52.9 percent) and *hlb* (20.9 percent) were widely present. *Hla* increased markedly across resistance gradients (34.3–85.7 percent), as shown in Fig. 1. These findings are consistent with prior reports showing that toxin-associated genes and resistance traits can be co-detected within the same isolate populations [1, 26]. The presence of *pvl* (8.0 percent), although infrequent overall, is notable because pvl-positive isolates were more frequently detected among MDR strains (Fig. 1). In the present dataset, this observation should be interpreted as a descriptive association rather than as evidence of enhanced pathogenicity or clonal behavior.

Pairwise analyses demonstrated significant enrichment of *hla* and *cna* across all resistance-category comparisons, while *fnbA* and *pvl* showed significance mainly in comparisons involving MDR isolates. In contrast, *fnbB* and *bbp* were not associated with resistance category (Fig. 1). While *mecA* showed the clearest association with multidrug resistance, statistically significant associations were also observed for selected virulence-associated genes. These findings should be interpreted cautiously as descriptive associations within the studied isolated population [9]. Limited statistical power, particularly for less frequently detected genes, may have reduced the ability to detect additional differences across resistance categories. Co-occurrence analysis identified recurrent co-detection patterns involving adhesion- and toxin-associated genes, particularly *fnbA-cna* and *hla-fnbA* (Table 4). These findings describe gene combinations present within the studied isolate population and do not establish functional interaction [23].

Higher-order gene clusters were rare and corresponded to isolates that simultaneously carried six or more virulence-associated genes. The clinical and epidemiological relevance of these combinations remains uncertain and requires further investigation in larger and longitudinal studies. A strength of this study is the combined evaluation of molecular virulence profiles and antimicrobial resistance patterns within a defined regional cohort. To our knowledge, this is among the first studies in Türkiye to describe both antimicrobial resistance patterns and selected virulence-associated genes in *S. aureus* isolates recovered from radicular cysts in a post-disaster regional setting. Previous work has seldom combined disaster context and comprehensive molecular profiling. These findings provide baseline molecular data that may support future epidemiological investigations in dental microbiology.

Characterizing virulence gene distributions in cyst-derived isolates provides descriptive information on isolate-level profiles in odontogenic infections. These observations may shape future investigations into diagnostic and management approaches for cases in which S. aureus is detected. Rapid PCR-based detection of mecA may support laboratory characterization of persistent odontogenic infections in which S. aureus is isolated, alongside routine phenotypic susceptibility testing [5]. Virulence gene profiling may contribute to epidemiological characterization of circulating strains, but it does not directly guide empirical antimicrobial therapy [12]. In this manuscript, the term “high-risk” should refer only to genes more frequently detected in MDR isolates in this dataset and should not be interpreted as a predictor of clinical outcome. These outcomes may be considered alongside efforts to strengthen microbiological monitoring and antibiotic stewardship in both routine and emergency dental care settings [29].

A formal a priori sample size calculation was not performed, as this study was designed as an exploratory, isolate-based molecular epidemiology investigation rather than a hypothesis-testing clinical trial.

This study has several limitations. First, its cross-sectional, isolate-based design supports only descriptive analysis. It cannot establish causal, temporal, or mechanistic relationships. Second, there was no pre-disaster comparison group or external control. The observed resistance and gene detection patterns cannot be linked to the disaster. Third, isolates came from a single regional center. This may limit generalizability. Fourth, no prior sample size calculation was performed. Statistical power may be low for rare genes and higher-order gene combinations. Finally, functional assays and genomic analyses were not performed. Gene detection should not be seen as direct evidence of gene expression, pathogenic behavior, or clinical impact. All these findings are descriptive. They are based on a single-center, cross-sectional dataset and do not support causal or predictive inferences.

## Conclusion

This cross-sectional, isolate-based molecular epidemiology study evaluated *S. aureus* isolates obtained from radicular cyst infections in Hatay, Türkiye, with respect to antimicrobial resistance patterns and selected virulence-associated genes. *MecA* positivity was more frequent in isolates with broader resistance categories, including multidrug-resistant (MDR) isolates.

Selected virulence-associated genes, including *hla, fnbA, cna*, and *pvl*, were more frequently detected among MDR isolates. These findings indicate co-detection of resistance traits and virulence-associated determinants within the same isolate collection.

Gene co-occurrence analysis revealed recurrent co-detection patterns among adhesion- and hemolysin-associated genes. Combinations involving adhesion- and hemolysin-associated genes, particularly *fnbA-cna, hla-fnbA*, and *hla-cna*, were most frequently observed, whereas higher-order combinations were uncommon.

The study design imposes important limitations. The cross-sectional structure, lack of a pre-disaster comparison group, sampling from a single geographic region, and absence of functional validation prevent causal interpretation. Temporal associations related to the post-disaster setting cannot be inferred. In addition, no prior sample size calculation was performed, which may reduce statistical power for less frequent gene combinations.

From a clinical microbiology standpoint, the findings may be relevant to combining molecular detection of major resistance markers, particularly mecA, with routine phenotypic susceptibility testing in persistent odontogenic infections in which S. aureus is isolated. Virulence gene profiling may assist epidemiological surveillance and strain characterization. It does not directly inform empirical antimicrobial selection.

Further multicenter studies, including genomic-level analyses and longitudinal sampling, are required to better define the epidemiological and clinical implications of the coexistence of antimicrobial resistance and virulence determinants.

## Supplementary Information

Supplementary Material 1.

Supplementary Material 2.

## Data Availability

De-identified data that support the findings of this study are available from the corresponding author upon reasonable request.

## Abbreviations

MDR: Multidrug-resistant
PCR: Polymerase chain reaction
CLSI: Clinical and Laboratory Standards Institute

## References

[1] Ahmad-Mansour N, Loubet P, Pouget C, Dunyach-Remy C, Sotto A, Lavigne JP, et al. *Staphylococcus aureus* toxins: an update on their pathogenic properties and potential treatments. Toxins Basel. 2021;13(10):677. 34678970 10.3390/toxins13100677 PMC8540901

[2] Ballhausen B, Kriegeskorte A, Schleimer N, Peters G, Becker K. The *mecA* homolog mecC confers resistance against β-lactams in *Staphylococcus aureus* irrespective of strain background. Antimicrob Agents Chemother. 2014;58(7):3791–8. 24752255 10.1128/AAC.02731-13 PMC4068569

[3] Baumgartner JC, Falkler WA. Bacteria in the apical 5 mm of infected root canals. J Endod. 1991;17(8):380–3. 1809801 10.1016/s0099-2399(06)81989-8

[4] Chambers HF, Deleo FR. Waves of resistance: *Staphylococcus aureus* in the antibiotic era. Nat Rev Microbiol. 2009;7(9):629–41. 19680247 10.1038/nrmicro2200 PMC2871281

[5] Clinical and Laboratory Standards Institute. Performance standards for antimicrobial susceptibility testing (33rd ed., CLSI supplement M100). Wayne: CLSI; 2023.

[6] Dimitrovski O, Pavlevska M. Etiopathogenetic aspects of radicular cysts. Balk J Dent Med. 2016;20(1):22–6.

[7] Du C, Wang Z, Lan D, Zhu R, Wang D, Wang H, et al. Clinical analysis of 1,038 cases of odontogenic jawbone cysts. BMC Oral Health. 2024;24(1):1387. 39548448 10.1186/s12903-024-05167-9 PMC11566165

[8] Farhan ZA, Jaber NN, Fayez RA. Investigation of antimicrobial resistance and virulence genes among methicillin-resistant Staphylococcus aureus from subclinical mastitic cows. J Anim Health Prod. 2025;13(Suppl 1):523-531. https://doi.org/10.17582/journal.jahp/2025/13.s1.523.531.

[9] Foster TJ, Geoghegan JA, Ganesh VK, Höök M. Adhesion, invasion and evasion: the many functions of *S. aureus* surface proteins. Nat Rev Microbiol. 2014;12(1):49–62. 24336184 10.1038/nrmicro3161 PMC5708296

[10] Furlan JPR, Sellera FP, Lincopan N, Debone D, Miraglia SGEK, Tavella RA. Catastrophic floods and antimicrobial resistance: interconnected threats. One Health. 2024;19:100891. 39310088 10.1016/j.onehlt.2024.100891 PMC11415860

[11] Kawada-Matsuo M, Komatsuzawa H. Factors affecting susceptibility of *S. aureus* to antibacterial agents. J Oral Biosci. 2012;54(2):86–9.

[12] Lakhundi S, Zhang K. Methicillin-resistant *S. aureus*: molecular characterization, evolution, and epidemiology. Clin Microbiol Rev. 2018;31(4):e00020-18. 30209034 10.1128/CMR.00020-18 PMC6148192

[13] Lina G, Piémont Y, Godail-Gamot F, Bes M, Peter MO, Gauduchon V, et al. PVL-positive *Staphylococcus aureus* in skin infections. Clin Infect Dis. 1999;29(5):1128–32. 10524952 10.1086/313461

[14] Martineau F, Picard FJ, Grenier L, Roy PH, Ouellette M, Bergeron MG. Multiplex PCR assays for detecting antibiotic resistance genes. J Antimicrob Chemother. 2000;46(4):527–34. 11020248 10.1093/jac/46.4.527

[15] Martineau F, Picard FJ, Roy PH, Ouellette M, Bergeron MG. Species-specific and ubiquitous-DNA-based assays for rapid identification of *Staphylococcus aureus*. J Clin Microbiol. 1998;36(3):618–23. 10.1128/JCM.36.3.618-623.1998. 9508283 10.1128/jcm.36.3.618-623.1998 PMC104596

[16] Mason WJ, Blevins JS, Beenken K, Wibowo N, Ojha N, Smeltzer MS. Multiplex PCR protocol for the diagnosis of staphylococcal infection. J Clin Microbiol. 2001;39(9):3332–8. 10.1128/JCM.39.9.3332-3338.2001. 11526172 10.1128/JCM.39.9.3332-3338.2001 PMC88340

[17] Mavrouli M, Mavroulis S, Lekkas E, Tsakris A. Impact of earthquakes on infectious diseases. Microorganisms. 2023;11(2):419. 36838384 10.3390/microorganisms11020419 PMC9968131

[18] Nappi F, Avtaar Singh SS. Host-bacterium interaction mechanisms in *S. aureus* endocarditis. Int J Mol Sci. 2023;24(13):11068. 37446247 10.3390/ijms241311068 PMC10341754

[19] Paharik AE, Horswill AR. The Staphylococcal Biofilm: Adhesins, Regulation, and Host Response. Microbiol Spectr. 2016;4(2):VMBF-0022-2015. 10.1128/microbiolspec.VMBF-0022-2015. 10.1128/microbiolspec.VMBF-0022-2015 PMC4887152 27227309

[20] Patti JM, Jonsson H, Guss B, Switalski LM, Wiberg K, Lindberg M, et al. Molecular characterization and expression of a gene encoding a *Staphylococcus aureus* collagen adhesin. J Biol Chem. 1992;267(7):4766–72. Erratum in: J Biol Chem, 269(15): 11672. 1311320

[21] Peacock SJ, Moore CE, Justice A, Kantzanou M, Story L, Mackie K, et al. Virulence gene combinations in *S. aureus*. Infect Immun. 2002;70(9):4987–96. 12183545 10.1128/IAI.70.9.4987-4996.2002 PMC128268

[22] Peng Q, Tang X, Dong W, Sun N, Yuan W. Biofilm formation and regulation in *S. aureus*. Antibiotics. 2023;12(1):12. 10.3390/antibiotics12010012 PMC9854888 36671212

[23] Schwermann N, Winstel V. Functional diversity of staphylococcal surface proteins. Front Microbiol. 2023;14:1196957. 37275142 10.3389/fmicb.2023.1196957 PMC10232760

[24] Seilie ES, Bubeck Wardenburg J. Pore-forming toxins of *S. aureus*. Semin Cell Dev Biol. 2017;72:101–16. 28445785 10.1016/j.semcdb.2017.04.003 PMC5823538

[25] Speziale P, Pietrocola G, Rindi S, Provenzano M, Provenza G, Di Poto A, et al. Structural role of *S. aureus* surface components. Future Microbiol. 2009;4(10):1337–52. 19995192 10.2217/fmb.09.102

[26] Tam K, Torres VJ. Staphylococcus aureus Secreted Toxins and Extracellular Enzymes. Microbiol Spectr. 2019;7(2):GPP3-0039-2018. 10.1128/microbiolspec.GPP3-0039-2018. 10.1128/microbiolspec.gpp3-0039-2018 PMC6422052 30873936

[27] Tong SYC, Davis JS, Eichenberger E, Holland TL, Fowler VG. *S. aureus* infections: epidemiology & management. Clin Microbiol Rev. 2015;28(3):603–61. 26016486 10.1128/CMR.00134-14 PMC4451395

[28] Turner NA, Sharma-Kuinkel BK, Maskarinec SA, Eichenberger EM, Shah PP, Carugati M, et al. MRSA research overview. Nat Rev Microbiol. 2019;17(4):203–18. 30737488 10.1038/s41579-018-0147-4 PMC6939889

[29] World Health Organization. Global report on antimicrobial resistance & emergency settings. Geneva: World Health Organization; 2023.

[30] Wu X, Wang H, Xiong J, Yang GX, Hu JF, Zhu Q, et al. Biofilm therapeutic strategies in *S. aureus*. Biofilm. 2024;7:100175. 38298832 10.1016/j.bioflm.2023.100175 PMC10827693

