# Supplementary material for: Co-detection of antimicrobial resistance and virulence-associated determinants in Staphylococcus aureus isolated from radicular cysts in a post-disaster region of Türkiye
Source: BMC Oral Health. 2026 May 13;26:1263. doi: 10.1186/s12903-026-08449-6 (PMC13371249; doi:10.1186/s12903-026-08449-6)
Supplement: Supplementary file 1 — Supplementary Material 1. [file 12903_2026_8449_MOESM1_ESM.docx]

**
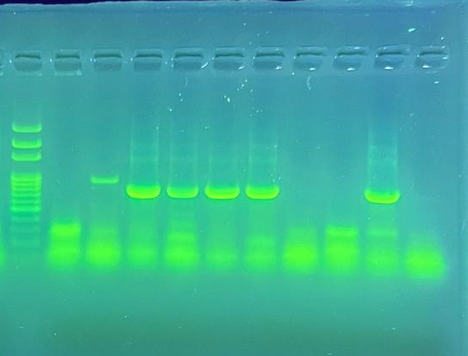
**

**M 1 2 3 4 5 6 7 8 9 10**

16S rRNA

(791 bp)

**Supplementary Figure S1.** PCR amplification of the *16S rRNA* gene (791 bp).

**Legend:** Agarose gel electrophoresis of 16S rRNA PCR products. M: 100 bp DNA ladder. Lanes 3-6 and 9 show positive amplification (791 bp). Lanes 1, 2, 7, 8 and 10 show no detectable amplification.

**
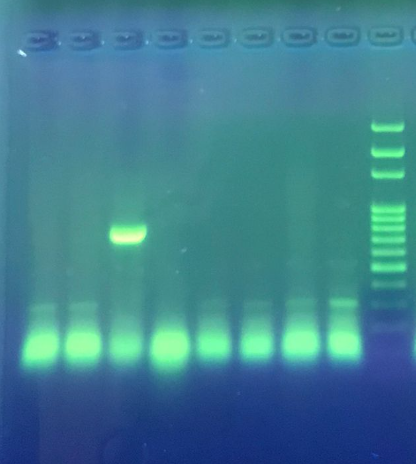
**

mecA

(331 bp)

**1 2 3 4 5 M**

**Supplementary Figure S2.** PCR amplification of the *mecA* gene (331 bp).

**Legend:** Agarose gel electrophoresis of mecA PCR products. M: 100 bp DNA ladder. Lanes 2-4 displayed 331 bp positive bands, while lane 1 showed no amplification.

**
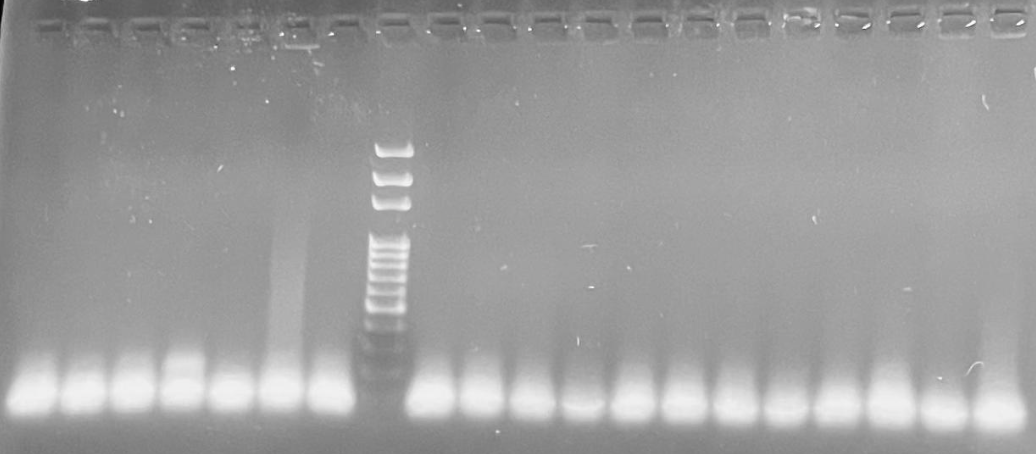
**

Hla

(209 bp)

**1 2 3 4 5 6 7 M**

**Supplementary Figure S3.** PCR amplification of the *hla* gene (209 bp).

**Legend:** Agarose gel electrophoresis of *hla* PCR products. M: 100 bp DNA ladder. Lanes 1-4 show positive 209 bp amplicons; lanes 5-7 show no amplification.

**
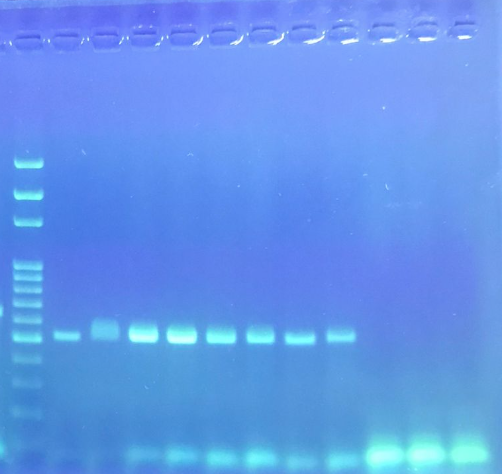
**

fnbA

(525 bp)

**M 1 2 3 4 5 6 7 8 9 10 11**

**Supplementary Figure S4.** PCR amplification of the *fnbA* gene (525 bp).

**Legend:** Agarose gel electrophoresis of fnbA PCR products. M: 100 bp DNA ladder. Lanes 1-8 exhibit positive amplification (525 bp), while lanes 9-11 show no amplification.


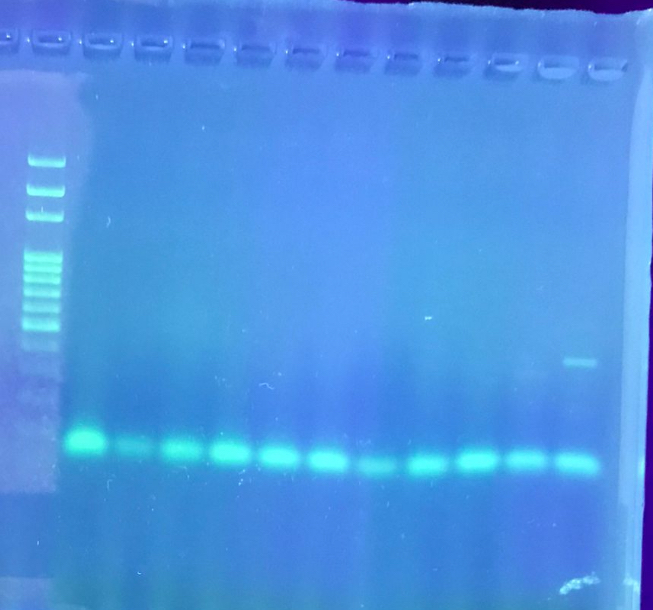


Pvl

(433 bp)

**M 1 2 3 4 5 6 7 8 9 10 11**

**Supplementary Figure S5.** PCR amplification of the *pvl* gene (433 bp).

**Legend:** Agarose gel electrophoresis of *pvl* PCR products. M: 100 bp DNA ladder. A positive 433 bp band was detected only in lane 6. Lanes 7-9 show no amplification.


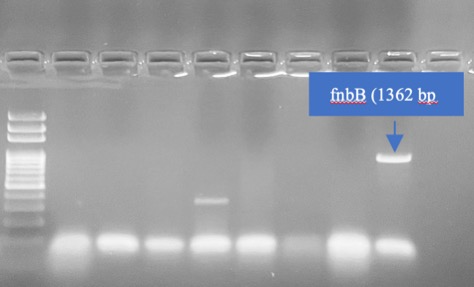


**Supplementary Figure S6.** Uncropped agarose gel image showing the *fnbB* amplicon (1362 bp).

**Legend:** Uncropped gel image displaying PCR amplification of the *fnbB* gene (1362 bp) on the upper gel. M: 100 bp DNA ladder. All visible bands correspond to the full-length amplicons prior to cropping for the main figure.

**
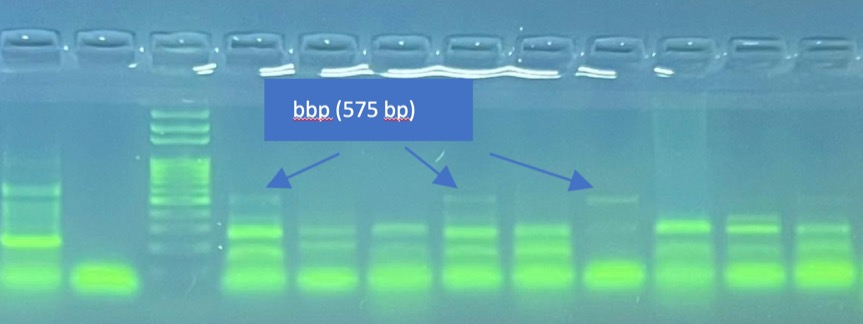
**

**Supplementary Figure S7.** Uncropped agarose gel image showing the *bbp* amplicon (575 bp).

**Legend:** Uncropped gel image demonstrating PCR amplification of the *bbp* gene (575 bp) on the upper gel. M: 100 bp DNA ladder. The figure shows the complete gel view used for the finalized cropped gel panel.


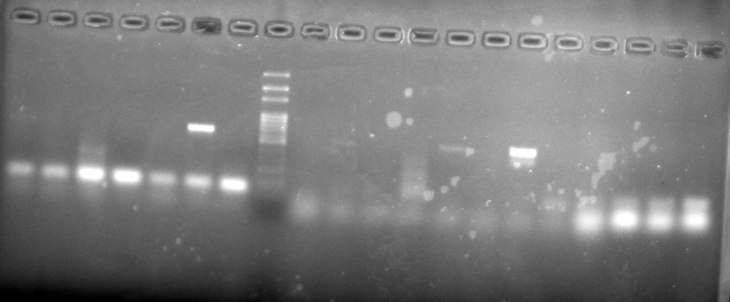


cna

(423 bp)

**1 2 3 4 5 6 7 M**

**Supplementary Figure S8.** PCR amplification of the *cna* gene (423 bp).

**Legend:** Agarose gel electrophoresis of *cna* PCR products. M: 100 bp DNA ladder; NK: negative control. Lanes 1-6 exhibited positive 423 bp amplicons, whereas lanes 7-9 showed no amplification.


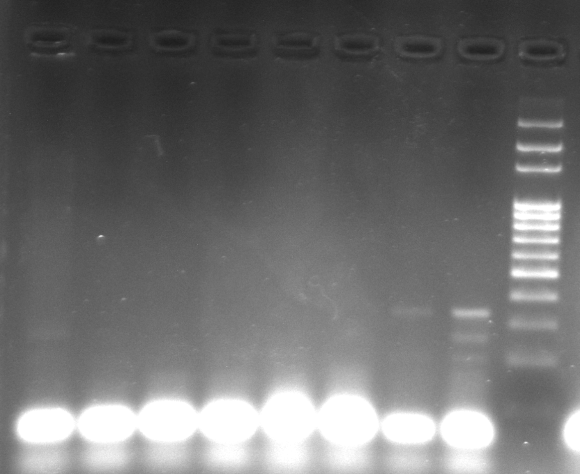


Hlb

(309 bp)

**1 2 3 4 5 6 7 8 M**

**Supplementary Figure S9.** PCR amplification of the *hlb* gene (309 bp).

**Legend:** Agarose gel electrophoresis of *hlb* PCR products. M: 100 bp DNA ladder. Positive amplification (309 bp) observed in lanes 1, 7 and 8; no amplification in lanes 2-6.


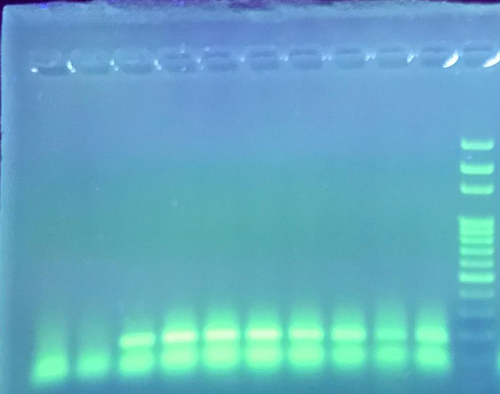


ebpS

(186 bp)

**1 2 3 4 5 6 7 8 9 10 M**

**Supplementary Figure S10.** PCR amplification of the *ebpS* gene (186 bp).

**Legend:** Agarose gel electrophoresis of *ebpS* PCR products. M: 100 bp DNA ladder. Positive bands (186 bp) were observed in lanes 3-10. No amplification was detected in lanes 1 and 2.
