# Supplementary material for: Co-detection of antimicrobial resistance and virulence-associated determinants in Staphylococcus aureus isolated from radicular cysts in a post-disaster region of Türkiye
Source: BMC Oral Health. 2026 May 13;26:1263. doi: 10.1186/s12903-026-08449-6 (PMC13371249; doi:10.1186/s12903-026-08449-6)
Supplement: Supplementary file 2 — Supplementary Material 2. [file 12903_2026_8449_MOESM2_ESM.docx]

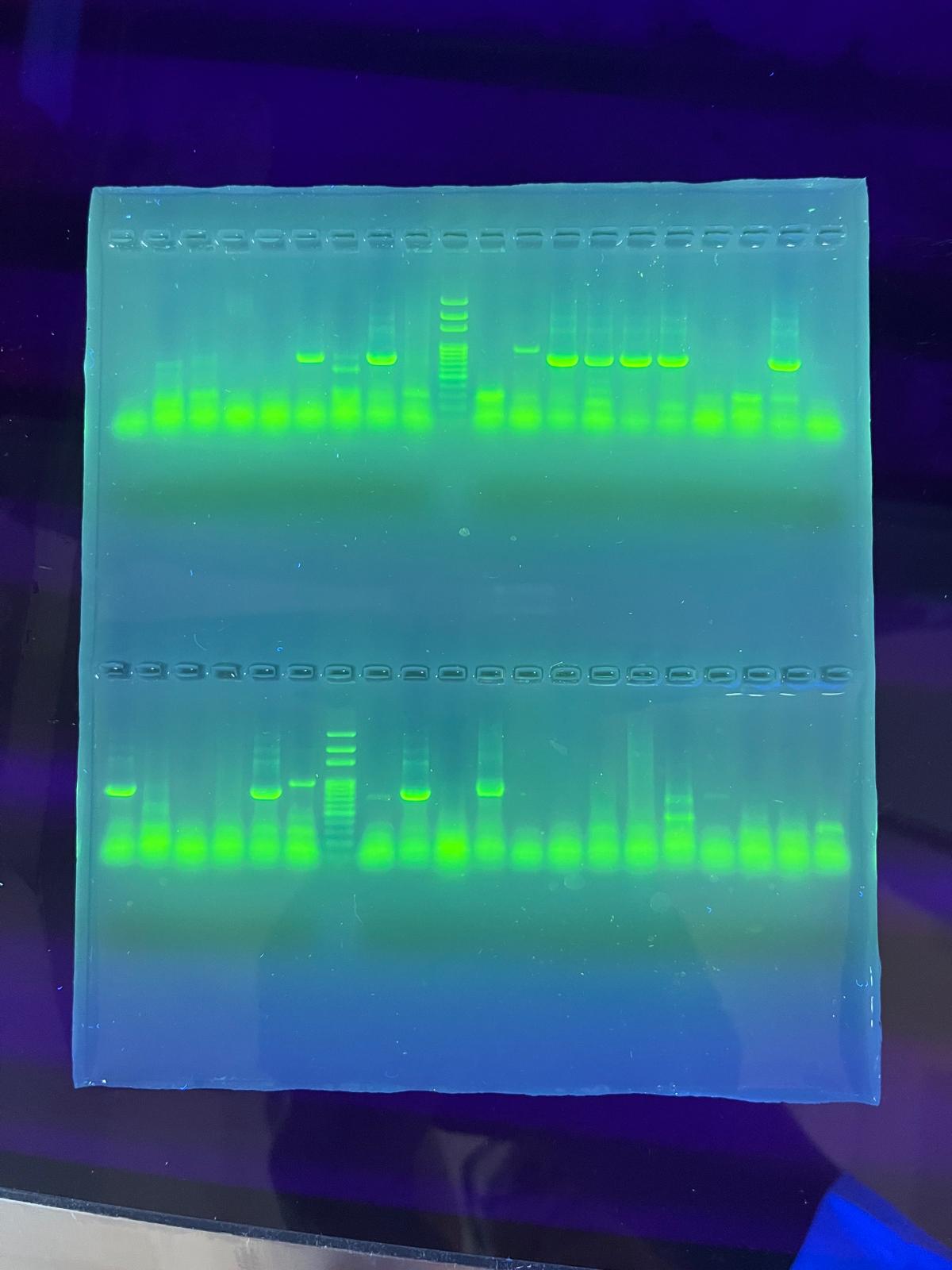


**Figure 1.** Uncropped gel image showing the 16S rRNA amplicon (791 bp, 100 bp DNA marker).


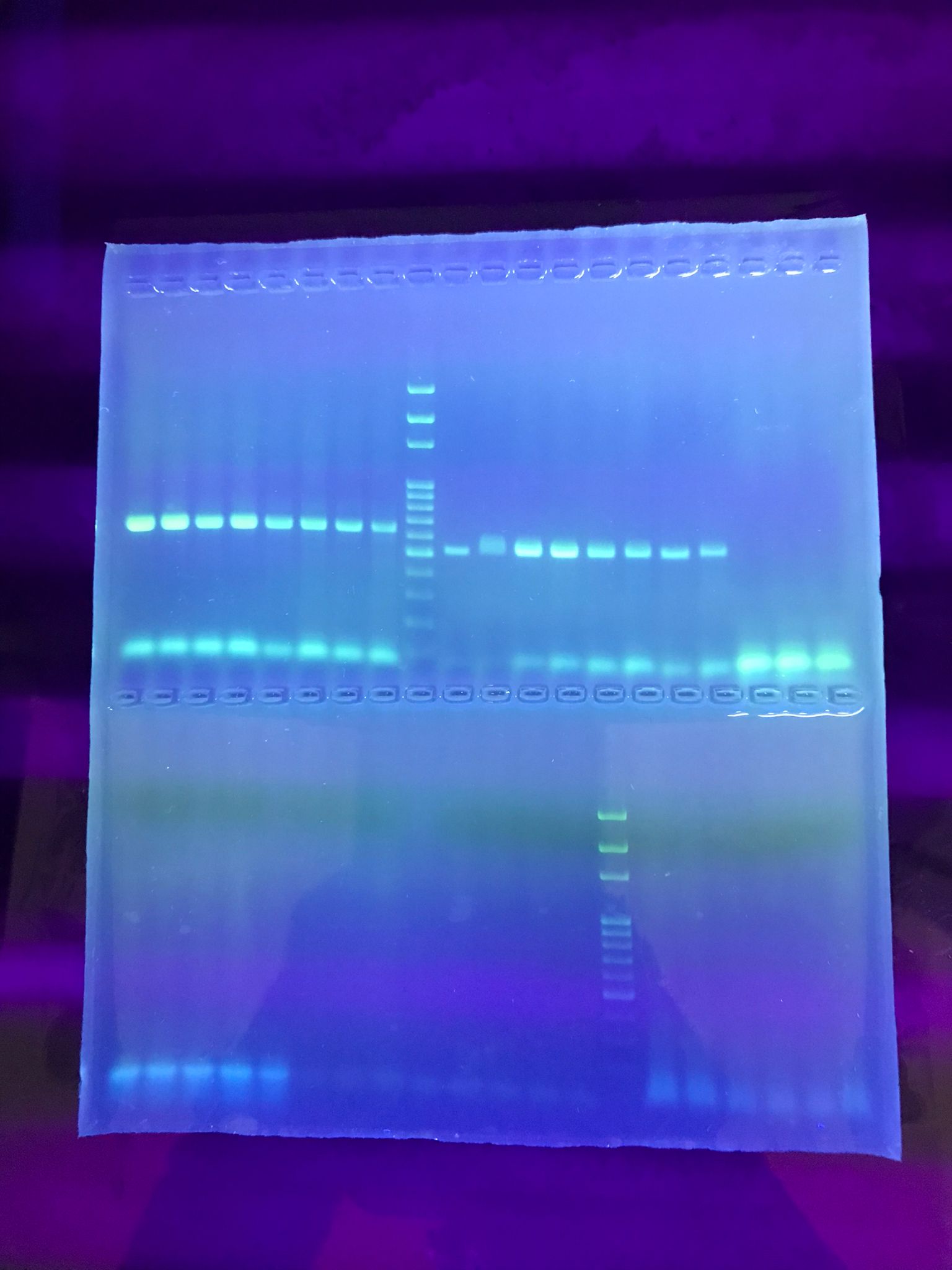


**Figure 2.** Uncropped gel image showing the the upper left band represents the clfA gene (638 bp), while the upper right band corresponds to the fnbA gene (525 bp).


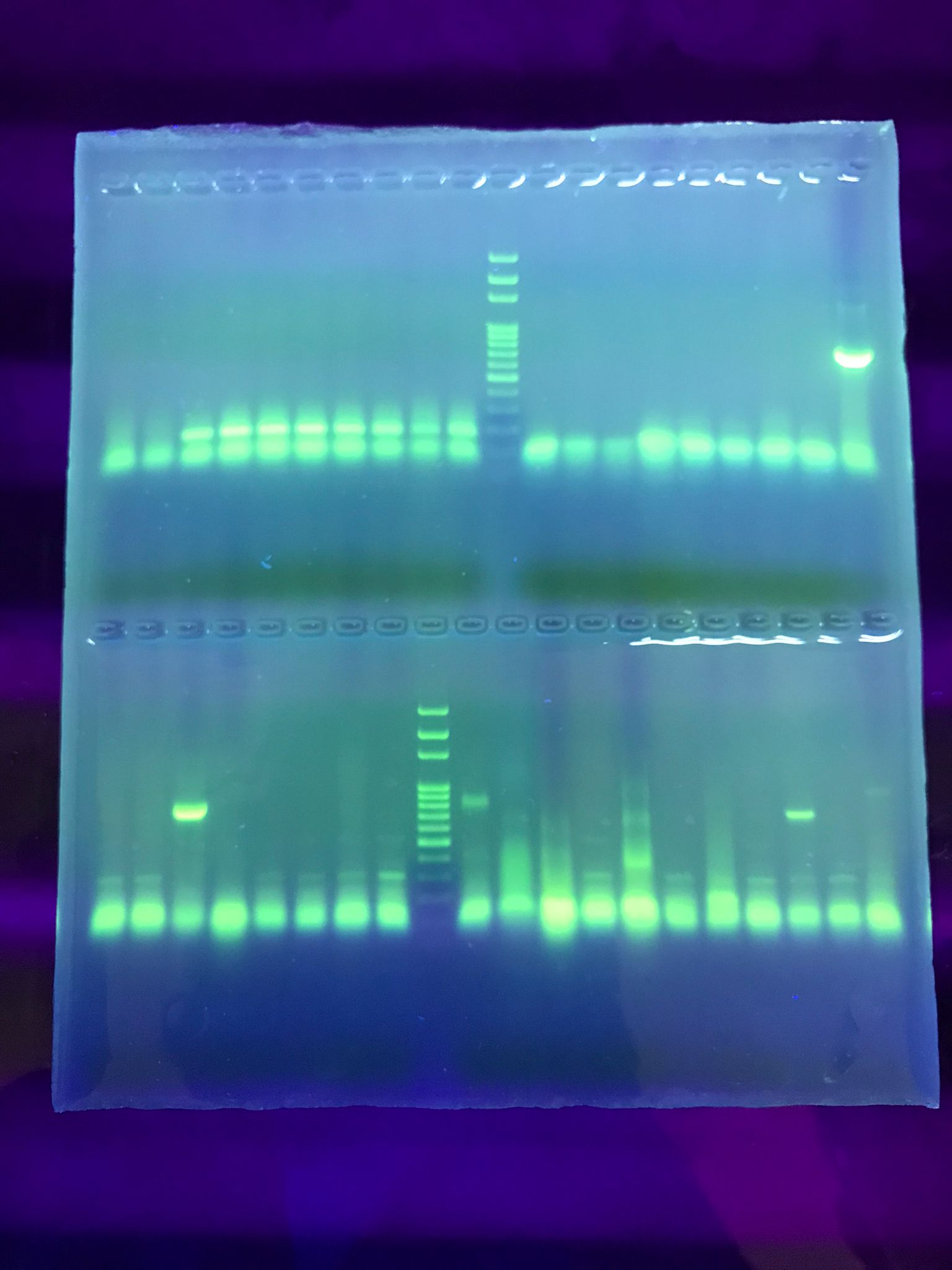


**Figure 3.** Uncropped gel image showing the ebps amplicon (186 bp, 100 bp DNA marker).


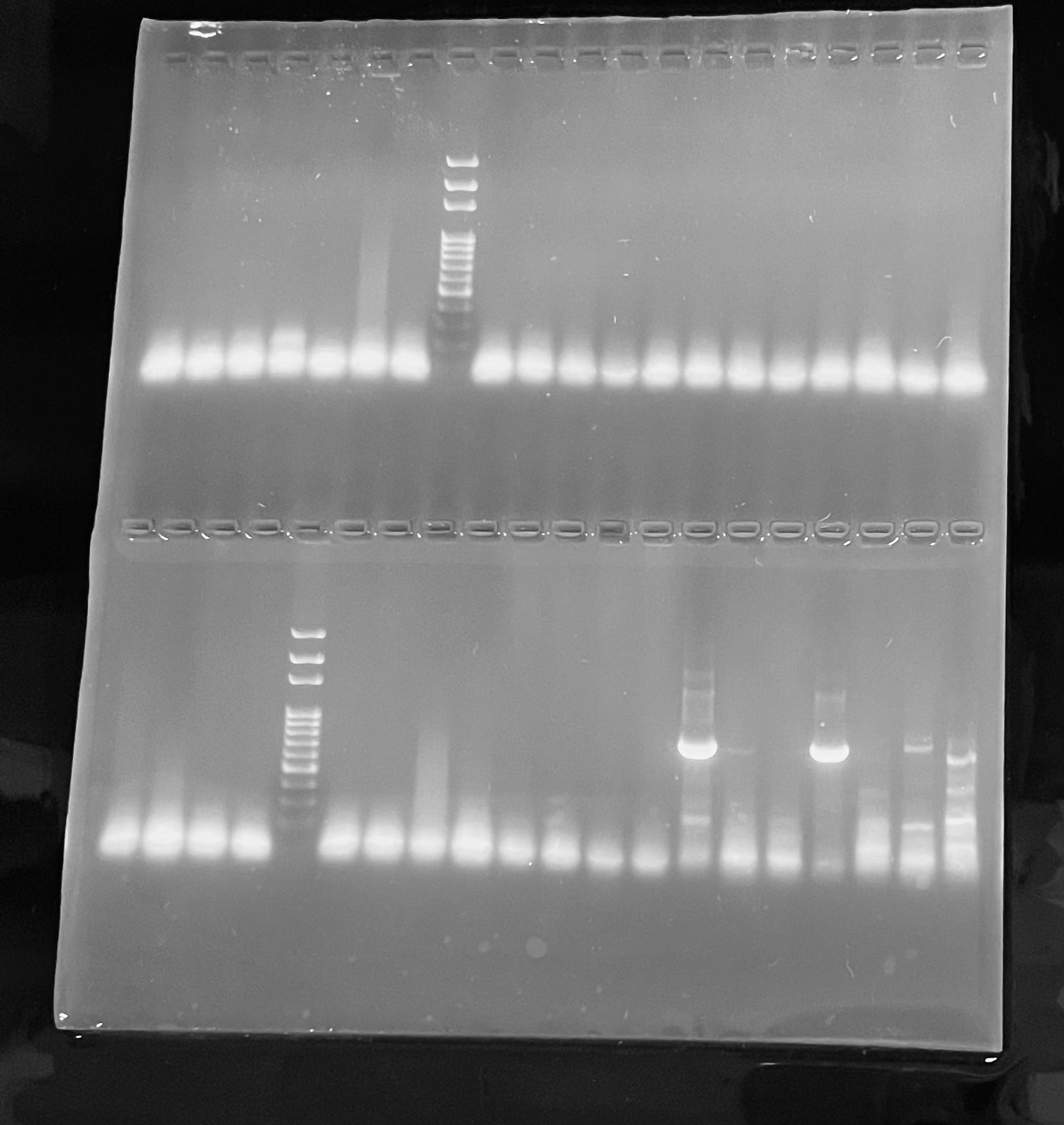


**Figure 4.** Uncropped gel image showing the hla amplicon (209 bp, 100 bp DNA marker).


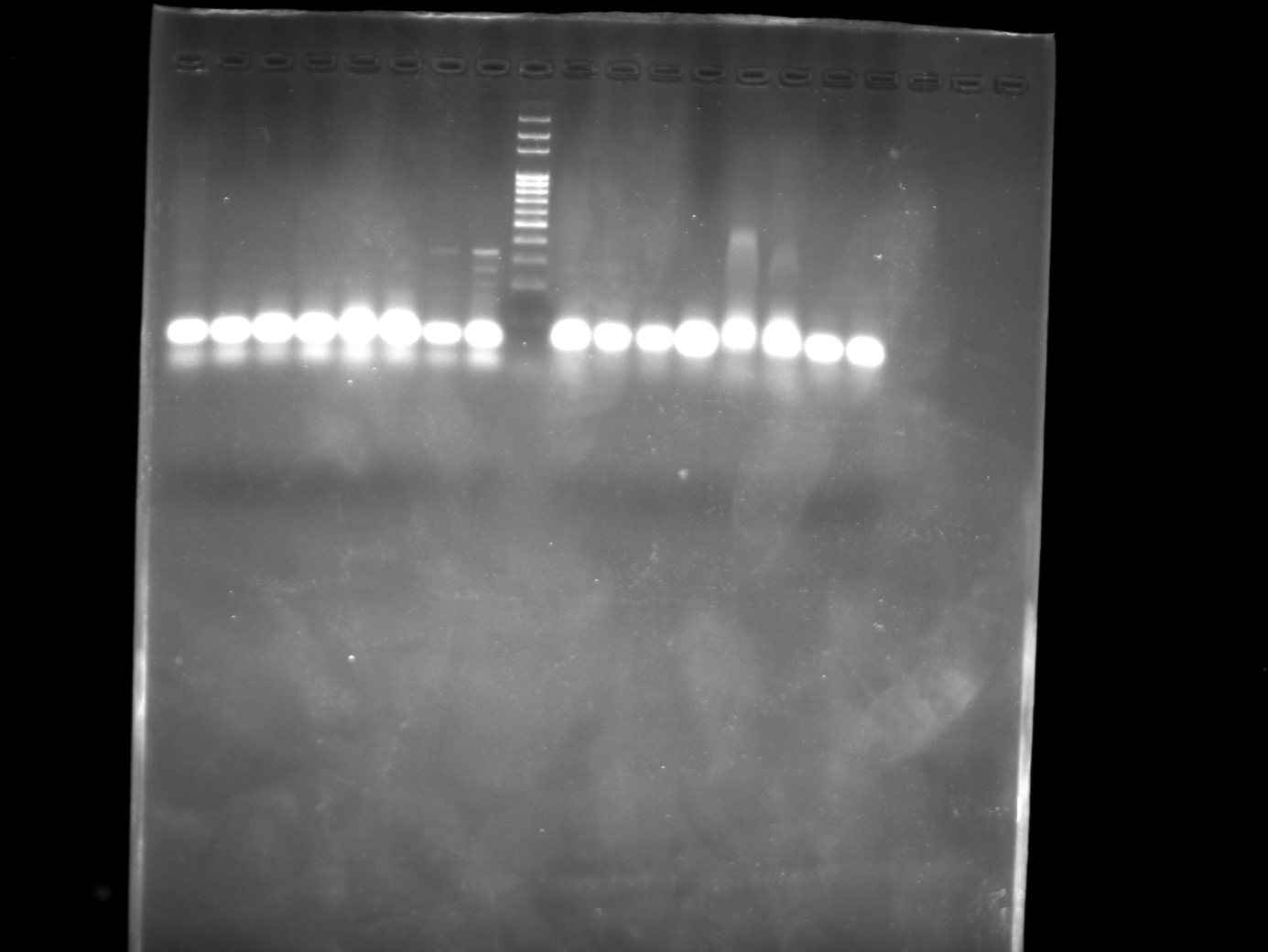


**Figure 5.** Uncropped gel image showing the hlb amplicon (309 bp, 100 bp DNA marker).


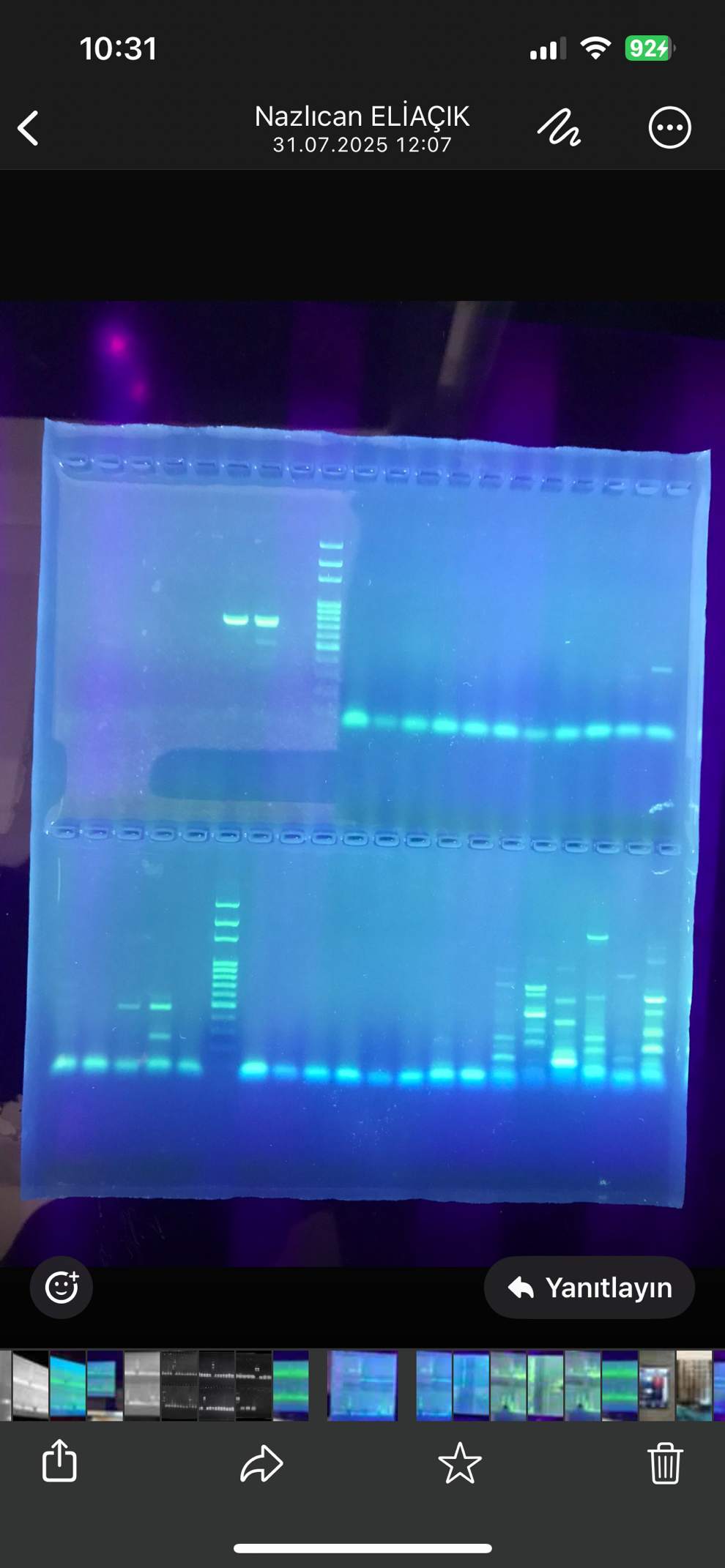


**Figure 6.** Uncropped gel image showing the pvl amplicon (433 bp, 100 bp DNA marker).


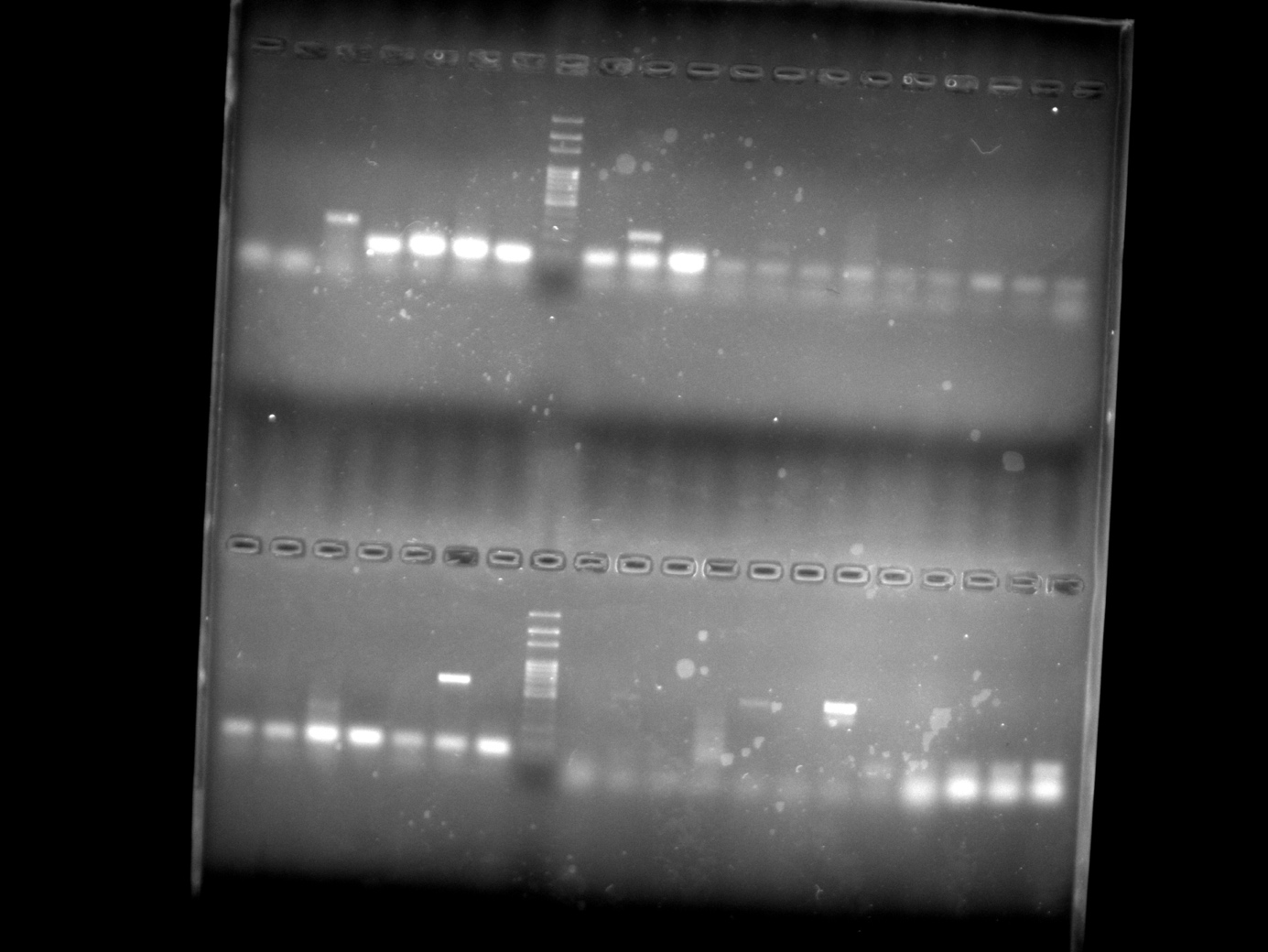


**Figure 7.** Uncropped gel image showing the hla amplicon (209 bp upper gel) cna amplicon (423 bp, lower gel) with the 100 bp marker.

**Figure 8.** Uncropped gel image showing the fnbB amplicon (1362 bp upper gel) with the 100 bp marker.

**Figure 9.** Uncropped gel image showing the bpb amplicon (575 bp upper gel) with the 100 bp marker.

**Figure 10.** Uncropped gel image showing the fnb amplicon (1362 bp upper gel) with the 100 bp marker.

**Figure 11.** Uncropped gel image showing the bnp amplicon (575 bp upper gel) with the 100 bp marker.
